# Supplementary material for: Validation of asthma recording in electronic health records: protocol for a systematic review
Source: BMJ Open. 2017 May 29;7(5):e014694. doi: 10.1136/bmjopen-2016-014694 (PMC5729974; doi:10.1136/bmjopen-2016-014694)
Supplement: Supplementary material 1 [file bmjopen-2016-014694supp001.pdf]

Appendix 1: Algorithm used for literature review

Asthma validation in electronic health records: a systematic review

MEDLINE

1 (validat* or verif*).mp. [mp=title, abstract, original title, name of substance word, subject heading word, keyword heading word, protocol supplementary concept word, rare disease supplementary concept word, unique identifier]

2 (PPV or PNV or NPV or "positive predictive value*" or "negative predictive value*" or "predictive positive value*" or "predictive negative value*" or "likelihood ratio" or precision or accuracy or "receiver operating characteristic*" or ROC or kappa).mp. [mp=title, abstract, original title, name of substance word, subject heading word, keyword heading word, protocol supplementary concept word, rare disease supplementary concept word, unique identifier]

3 Validation Studies/ or validation.mp. or Validation Studies as Topic/

4 (electronic* or digital* or computeri?ed or programmed or automated or database or data base).mp. [mp=title, abstract, original title, name of substance word, subject heading word, keyword heading word, protocol supplementary concept word, rare disease supplementary concept word, unique identifier]

5 asthma.mp. or Asthma/ or Asthma, Occupational/ or Asthma, Exercise-Induced/

6 Database Management Systems/

7 1 or 2 or 3

8 4 or 6

9 5 and 7 and 8

EMBASE

1 (validat* or verif*).mp. [mp=title, abstract, heading word, drug trade name, original title, device manufacturer, drug manufacturer, device trade name, keyword]

2 validation.mp. or validation study/ or validation process/

3 (sensitivity or specificity or "Sensitivity and Specificity").mp. [mp=title, abstract, heading word, drug trade name, original title, device manufacturer, drug manufacturer, device trade name, keyword]

4 (PPV or PNV or NPV or "positive predictive value" or "predictive negative value" or "negative predictive value" or "likelihood ratio" or precision or accuracy or "receiver operating characteristic" or ROC or kappa).mp. [mp=title, abstract, heading word, drug trade name, original title, device manufacturer, drug manufacturer, device trade name, keyword, floating subheading]

5 (electronic* or digital* or computeri?ed or programmed or automated or database or data base).mp. [mp=title, abstract, heading word, drug trade name, original title, device manufacturer, drug manufacturer, device trade name, keyword]

6 mild persistent asthma/ or nocturnal asthma/ or experimental asthma/ or moderate persistent asthma/ or severe persistent asthma/ or Asthma.mp. or exercise induced asthma/ or occupational asthma/ or intrinsic asthma/ or asthma/ or allergic asthma/ or extrinsic asthma/ or mild intermittent asthma/

7 1 or 2 or 3 or 4

8 5 and 6 and 7
