# Supplementary material for: Validation of asthma recording in electronic health records: protocol for a systematic review
Source: BMJ Open. 2017 May 29;7(5):e014694. doi: 10.1136/bmjopen-2016-014694 (PMC5729974; doi:10.1136/bmjopen-2016-014694)
Supplement: Author's manuscript [file bmjopen-2016-014694.draft_revisions.pdf]

BMJ Open

Validation of asthma recording in electronic health records: protocol for a systematic review

Journal:	*BMJ Open*
Manuscript ID	bmjopen-2016-014694
Article Type:	Protocol
Date Submitted by the Author:	11-Oct-2016
Complete List of Authors:	Nissen, Francis; London School of Hygiene and Tropical Medicine, EPH - ENCD<br>Quint, Jennifer; Imperial College London, Respiratory Epidemiology, Occupational Medicine and Public Health<br>Wilkinson, Samantha; London School of Hygiene and Tropical Medicine, Epidemiology<br>Müllerová, Hana; GlaxoSmithKline, Epidemiology Department<br>Smeeth, Liam; London School of Hygiene and Tropical Medicine, Epidemiology and Population Health<br>Douglas, Ian; London School of Hygiene and Tropical Medicine, Epidemiology and Population Health
**Primary Subject Heading**:	Epidemiology
Secondary Subject Heading:	Respiratory medicine, Health informatics, Health services research
Keywords:	Asthma < THORACIC MEDICINE, Validation, Electronic Health Records, Sensitivity, Positive Predictive Value, Negative Predictive Value

SCHOLARONE™
Manuscripts

Validation of asthma recording in electronic health records: protocol for a systematic review

Authors: Francis Nissen,[1] Jennifer Quint,[2] Samantha Wilkinson,[1] Hana Mullerova,[3] Liam Smeeth,[1] Ian Douglas[1]

[1]Department of Non-Communicable Disease Epidemiology, London School of Hygiene and Tropical Medicine, London, UK; [2]National Heart and Lung Institute, Imperial College, London, UK; RWD & Epidemiology, GSK R&D , Uxbridge , UK

Correspondence:

Francis Nissen, MD, MSc, Department of Non-Communicable Disease Epidemiology, London School of Hygiene and Tropical Medicine, London, UK.

Address: Keppel Street, London, WC1E 7HT, UK

E-mail: francis.nissen@lshtm.ac.uk

For peer review only - http://bmjopen.bmj.com/site/about/guidelines.xhtml

ABSTRACT

Background: Asthma is a common, heterogeneous disease with significant morbidity and mortality worldwide. It can be difficult to define in epidemiological studies using electronic health records, as the diagnosis is based on non-specific respiratory symptoms and spirometry, neither of which are routinely registered. Electronic health records can nonetheless be valuable to study the epidemiology, management, health-care utilization and control of asthma. For health databases to be useful sources of information, asthma diagnoses should ideally be validated. The primary objectives are to provide an overview of the methods used to validate asthma diagnoses in electronic health records and the results of the validation studies.

The primary objective of this systematic review is to summarize definitions and test measures estimates of validated asthma diagnoses recorded in electronic health record databases.

Methods: EMBASE and MEDLINE will be systematically searched for appropriate search terms. The searches will cover all studies up to October 2016 and will yield studies that have validated algorithms or codes for the diagnosis of asthma in electronic health records. To be included, a nationally/internationally recognised standard case definition of asthma, has to be used and at least one test validation measure (sensitivity, specificity, positive predictive value, negative predictive value or other) has to be present. Two independent reviewers will summarise key data in a uniform table and assess study quality using the Standards for

For peer review only

For peer review only - http://bmjopen.bmj.com/site/about/guidelines.xhtml

Reporting of Diagnostic Accuracy (STARD) criteria.

Ethics and dissemination: This study is a synthesis of previously published studies and, therefore, no ethical approval is required. The results will be submitted to a peer-reviewed journal for publication. Results from this systematic review can be used to study outcome research on asthma and can be used to identify case definitions for asthma.

Trial registration number

The protocol has been registered in the PROSPERO database with registration number CRD42016041798.

Keywords

Asthma, Validation, Electronic Health Records, Sensitivity, Specificity, Positive Predictive Value, Negative Predictive Value

For peer review only

Strengths and limitations of this study

- To our knowledge, this is the first systematic review to identify and evaluate methods used to validate a recording of asthma diagnosis in electronic health records.
- The review of validation of asthma diagnosis codes in electronic health records is going to inform selection of asthma definitions used by future health outcome studies and identify any gaps in quality and scope of validation studies. It will also provide an overview of the standard case definitions and algorithms with their PPV, NPV, sensitivity or specificity.
- Different databases may validate different algorithms to identify asthma, which might limit the generalisability of these algorithms as they are context-specific.
- This review is focused on the methodology of asthma recording validation, and not on all outcome results of studies (except the validation results). Because of this, publication bias might be an issue (methods that do not find positive results may be less likely to have been published).

BACKGROUND

Asthma is a common chronic inflammatory disease of the airways. This condition is characterised by a variable expiratory airflow limitation which is generally reversible. The core symptoms are cough, wheeze, breathlessness and chest tightness [1]. Asthma episodes can range from mild attacks, which interrupt daily life and work productivity, to severe and

For peer review only

For peer review only - http://bmjopen.bmj.com/site/about/guidelines.xhtml

life-threatening attacks [2]. Asthma is inherently variable and individuals will experience fluctuating symptoms. Most commonly, asthma emerges in childhood, but it can also arise in adulthood. Therefore, adult asthma consists of both persistent or relapsed childhood disease and true incident adult disease. There is no cure, but with the right treatment, symptoms can usually be managed and asthma patients can lead their lives without disruption [1].

The widespread adoption of electronic health records (EHR) means that large population-based primary and secondary care databases are available, proving a great opportunity for research on asthma and other diseases. The availability of routinely generated longitudinal records for research has dramatically increased over the last decades [3]. However, the primary function of electronic health records is to support healthcare clinical decision making, not research purposes. The integrity of the research generated from EHR's may be questionable, unless data are thoroughly validated for this purpose [4-7].

EHRs store information about diagnoses as clinical codes. One can ascertain a diagnosis from EHRs using either a single code or an algorithm consisting of 2 or more components (9,10). The most common method to assess the validity of coding is to compare them to a gold standard such as another linkable dataset or request a verification from the treating physician or the patient via a questionnaire [8]. Another approach is active case detection where the databases are constantly screened to identify cases that emerge [9].

For peer review only

For peer review only - http://bmjopen.bmj.com/site/about/guidelines.xhtml

Several limitations apply to the validation of diagnosis recording in EHRs. First, individual databases often only cover a single care setting (primary or secondary care), as such case ascertainment only relies on a partial description of the healthcare pathway [10]. Another issue is that the validity of different diseases will not necessarily be the same in a given dataset. For example, mental health disorders such as anxiety or depression might be coded using less specific symptoms, whereas the validity of diagnoses with a very high specificity such as breast cancer is likely to be superior. There have been multiple studies which have measured the validity of specific databases for asthma [11 12]. Sharifi et al have conducted a systematic review on validated methods to capture acute bronchospasm using administrative or claims data [13], which yielded two validation studies of bronchospasm codes [14 15].

This systematic literature review aims to evaluate validation studies of codes or algorithms aimed to identify cases of asthma in specifically in electronic health records. Such a study has not been yet published in the medical literature, to the best of our knowledge.

For peer review only

Research question

The primary objectives of this systematic review are to provide an overview of both the methods with which asthma diagnosis recording has been validated in electronic health records and the estimates of the validation test measures.

The questions of interest for this systematic review are:

For peer review only - http://bmjopen.bmj.com/site/about/guidelines.xhtml

A. Which electronic health record databases have been used to obtain information on the diagnosis of asthma?

B. Which algorithms or codes have been used to define an asthma diagnosis (including diagnostic codes, possible spirometry tests and clinical descriptions)?

C. How were the diagnostic criteria applied to the data sources and which other approaches have been used to validate a case definition?

D. What are the estimates for the PPV, NPV, specificity and sensitivity for a diagnosis of asthma in an electronic health records?

METHODS

MEDLINE and EMBASE will be searched for the terms “asthma”, “validation”, “electronic databases” and synonyms for each of these terms. In addition, reference lists of review articles and retrieved articles will be reviewed. The PRISM flow diagram can be found in figure 1 and the search strategy can be found in the appendix.

Inclusion criteria

Any type of observational study design that used electronic health records to validate the recording of a diagnosis of asthma will be considered. Articles will only be considered if published in English and before October 2016. Usage of a reference standard case definition for asthma diagnosis, such as the definition of asthma outlined by the Global Initiative for Asthma [2], and the presence of at least one test measure (PPV, sensitivity or others) will be

For peer review only

For peer review only - http://bmjopen.bmj.com/site/about/guidelines.xhtml

necessary for inclusion.

Exclusion criteria

Studies which involve pharmacovigilance databases (signal detection or spontaneous reporting), studies without validation process of asthma recording, non-population-based studies, and conference abstracts will be excluded. Claims databases will also be excluded, as a systematic review on the validated methods to capture acute bronchospasm using claims data has been published recently [13].

Two independent authors will scan the abstracts and titles against the research questions and exclusion criteria and select articles for full-text review. After this full-text article review, eligibility for inclusion in the report will be decided by consensus or arbitration by a third reviewer. A uniform table with information of each included study will be populated after data extraction, which will include information on the author, date of publication, journal, database, tested codes, algorithms, population, gold standard and test measure(s).

The overall quality, methods and design of the included studies will be examined using a checklist created by Benchimol et al [16], which can be found in the appendix. This checklist is based on the criteria of the Standards for Reporting of Diagnostic accuracy (STARD) initiative [17].

Data synthesis

The methods for asthma recording validation will be summarised in a narrative synthesis and

For peer review only - http://bmjopen.bmj.com/site/about/guidelines.xhtml

tables describing all identified verification processes, and their results. These results will consist of the recorded PPV, NPV, sensitivity and specificity of the included studies. Where possible, these tests will be calculated if they are not reported within the study.

Dissemination and ethics

This study is a synthesis of previously published studies, so no ethical approval is required. The protocol has been registered in the PROSPERO database with registration number CRD42016041798. The results will be submitted for publication and will be disseminated through research conferences and peer reviewed journals.

Competing interests

FN and SW are funded by a GSK scholarship during their PhD programs.

Contributors

JQ, ID, LS and HM were responsible for developing the research question and have advised on the data collection and search strategies. FN drafted the manuscript, FN and SW will review the literature and summarise the found papers. ID is responsible for study management and coordination. All authors have read, commented on and approved the final manuscript.

Data sharing statement

For peer review only
For peer review only - http://bmjopen.bmj.com/site/about/guidelines.xhtml

Study data will be available on request to FN once the research team has completed pre-planned analyses.

References

1. WHO. Asthma Fact sheet No307. Secondary Asthma Fact sheet No307 2013.
2. GINA. Global Strategy for Asthma Management and Prevention 2016. Available from: www.ginasthma.org: Global Initiative for Asthma, 2016.
3. Langan SM, Benchimol EI, Guttmann A, et al. Setting the RECORD straight: developing a guideline for the REporting of studies Conducted using Observational Routinely collected Data. Clinical epidemiology 2013;**5**:29-31 doi: 10.2147/clep.s36885[published Online First: Epub Date]|.
4. Denney MJ, Long DM, Armistead MG, Anderson JL, Conway BN. Validating the extract, transform, load process used to populate a large clinical research database. International journal of medical informatics 2016;**94**:271-4 doi: 10.1016/j.ijmedinf.2016.07.009[published Online First: Epub Date]|.
5. Rimland JM, Abraha I, Luchetta ML, et al. Validation of chronic obstructive pulmonary disease (COPD) diagnoses in healthcare databases: a systematic review protocol. BMJ open 2016;**6**(6):e011777 doi: 10.1136/bmjopen-2016-011777[published Online First: Epub Date]|.
6. Abraha I, Giovannini G, Serraino D, Fusco M, Montedori A. Validity of breast, lung and colorectal cancer diagnoses in administrative databases: a systematic review protocol. BMJ open 2016;**6**(3):e010409 doi: 10.1136/bmjopen-2015-010409[published Online First: Epub Date]|.
7. Lo Re V, 3rd, Haynes K, Forde KA, Localio AR, Schinnar R, Lewis JD. Validity of The Health Improvement Network (THIN) for epidemiologic studies of hepatitis C virus infection. Pharmacoepidemiology and drug safety 2009;**18**(9):807-14 doi: 10.1002/pds.1784[published Online First: Epub Date]|.
8. Weiskopf NG, Weng C. Methods and dimensions of electronic health record data quality assessment: enabling reuse for clinical research. Journal of the American Medical Informatics Association : JAMIA 2013;**20**(1):144-51 doi: 10.1136/amiajnl-2011-000681[published Online First: Epub Date]|.
9. Wing K, Douglas I, Bhaskaran K, et al. Development of predictive genetic tests for improving the safety of new medicines: the utilization of routinely collected electronic health records. Drug discovery today 2014;**19**(4):361-6 doi: 10.1016/j.drudis.2013.11.003[published Online First: Epub Date]|.
10. Wing K, Bhaskaran K, Smeeth L, et al. Optimising case detection within UK electronic health records: use of multiple linked databases for detecting liver injury. BMJ open

For peer review only

For peer review only - http://bmjopen.bmj.com/site/about/guidelines.xhtml

2016;**6**(9):e012102 doi: 10.1136/bmjopen-2016-012102[published Online First: Epub Date]|.

11. Xi N, Wallace R, Agarwal G, Chan D, Gershon A, Gupta S. Identifying patients with asthma in primary care electronic medical record systems: Chart analysis–based electronic algorithm validation study. Canadian Family Physician 2015;**61**(10):e474-e83
12. Cornish RP, Henderson J, Boyd AW, Granell R, Van Staa T, Macleod J. Validating childhood asthma in an epidemiological study using linked electronic patient records. BMJ open 2014;**4**(4):e005345 doi: 10.1136/bmjopen-2014-005345[published Online First: Epub Date]|.
13. Sharifi M, Krishanswami S, McPheeters ML. A systematic review of validated methods to capture acute bronchospasm using administrative or claims data. Vaccine 2013;**31 Suppl 10**:K12-20 doi: 10.1016/j.vaccine.2013.06.091[published Online First: Epub Date]|.
14. Mullooly JP, Schuler R, Mesa J, Drew L, DeStefano F. Wheezing lower respiratory disease and vaccination of premature infants. Vaccine 2011;**29**(44):7611-7 doi: 10.1016/j.vaccine.2011.08.022[published Online First: Epub Date]|.
15. Sanders DL, Gregg W, Aronsky D. Identifying asthma exacerbations in a pediatric emergency department: a feasibility study. International journal of medical informatics 2007;**76**(7):557-64 doi: 10.1016/j.ijmedinf.2006.03.003[published Online First: Epub Date]|.
16. Benchimol EI, Manuel DG, To T, Griffiths AM, Rabeneck L, Guttmann A. Development and use of reporting guidelines for assessing the quality of validation studies of health administrative data. Journal of clinical epidemiology 2011;**64**(8):821-9 doi: 10.1016/j.jclinepi.2010.10.006[published Online First: Epub Date]|.
17. Bossuyt PM, Reitsma JB, Bruns DE, et al. Towards complete and accurate reporting of studies of diagnostic accuracy: the STARD initiative. Family practice 2004;**21**(1):4-10

For peer review only

For peer review only - http://bmjopen.bmj.com/site/about/guidelines.xhtml

Figure legends

Figure 1. PRISMA Flow Diagram

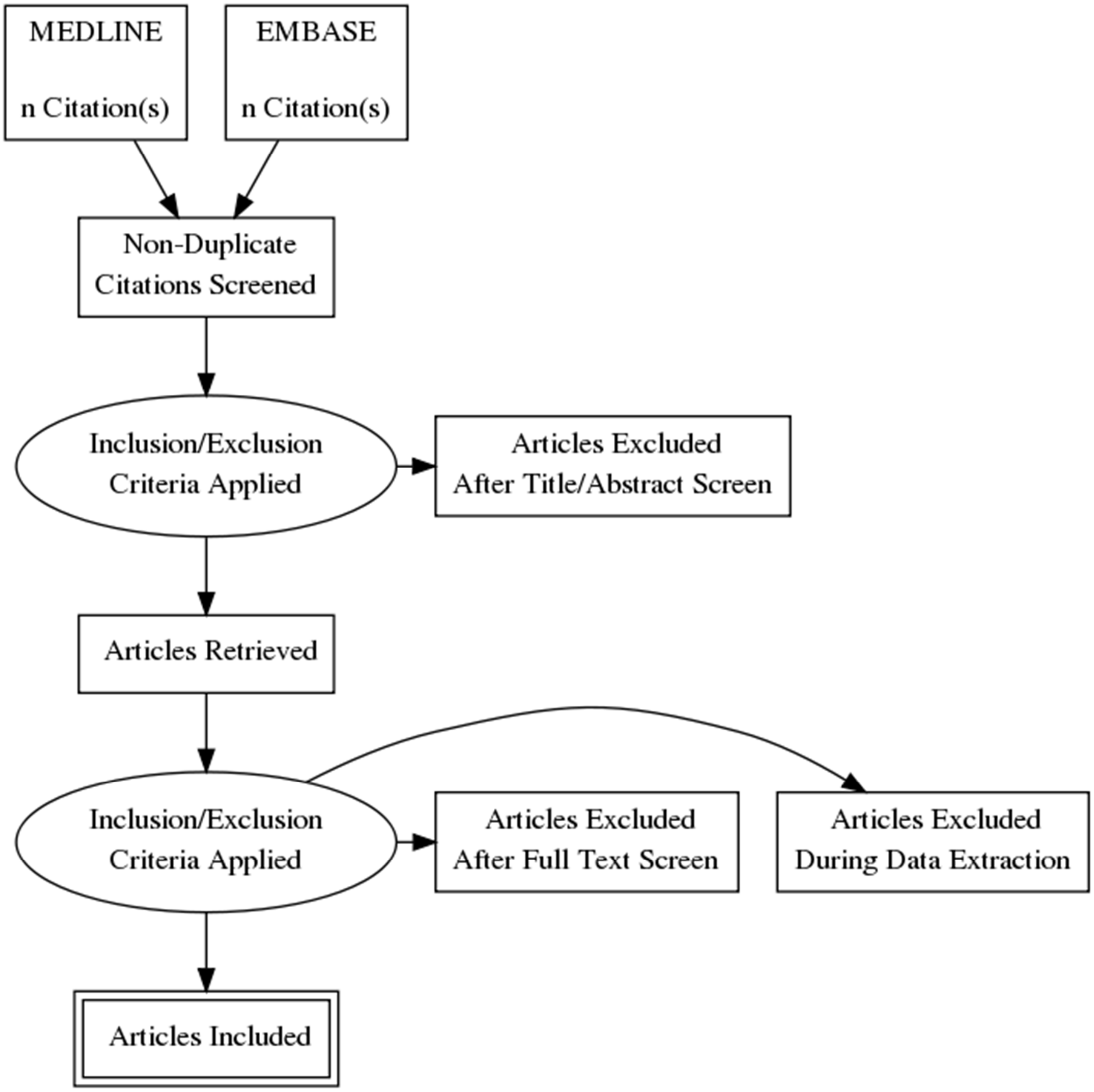

Appendix 1: Algorithm used for literature review

Asthma validation in electronic health records: a systematic review

MEDLINE

1 (validat* or verif*).mp. [mp=title, abstract, original title, name of substance word, subject heading word, keyword heading word, protocol supplementary concept word, rare disease supplementary concept word, unique identifier]

2 (PPV or PNV or NPV or "positive predictive value*" or "negative predictive value*" or "predictive positive value*" or "predictive negative value*" or "likelihood ratio" or precision or accuracy or "receiver operating characteristic*" or ROC or kappa).mp. [mp=title, abstract, original title, name of substance word, subject heading word, keyword heading word, protocol supplementary concept word, rare disease supplementary concept word, unique identifier]

3 Validation Studies/ or validation.mp. or Validation Studies as Topic/

4 (electronic* or digital* or computeri?ed or programmed or automated or database or data base).mp. [mp=title, abstract, original title, name of substance word, subject heading word, keyword heading word, protocol supplementary concept word, rare disease supplementary concept word, unique identifier]

5 asthma.mp. or Asthma/ or Asthma, Occupational/ or Asthma, Exercise-Induced/

6 Database Management Systems/

7 1 or 2 or 3

8 4 or 6

9 5 and 7 and 8

For peer review only - http://bmjopen.bmj.com/site/about/guidelines.xhtml

EMBASE

1 (validat* or verif*).mp. [mp=title, abstract, heading word, drug trade name, original title, device manufacturer, drug manufacturer, device trade name, keyword]

2 validation.mp. or validation study/ or validation process/

3 (sensitivity or specificity or "Sensitivity and Specificity").mp. [mp=title, abstract, heading word, drug trade name, original title, device manufacturer, drug manufacturer, device trade name, keyword]

4 (PPV or PNV or NPV or "positive predictive value" or "predictive negative value" or "negative predictive value" or "likelihood ratio" or precision or accuracy or "receiver operating characteristic" or ROC or kappa).mp. [mp=title, abstract, heading word, drug trade name, original title, device manufacturer, drug manufacturer, device trade name, keyword, floating subheading]

5 (electronic* or digital* or computeri?ed or programmed or automated or database or data base).mp. [mp=title, abstract, heading word, drug trade name, original title, device manufacturer, drug manufacturer, device trade name, keyword]

6 mild persistent asthma/ or nocturnal asthma/ or experimental asthma/ or moderate persistent asthma/ or severe persistent asthma/ or Asthma.mp. or exercise induced asthma/ or occupational asthma/ or intrinsic asthma/ or asthma/ or allergic asthma/ or extrinsic asthma/ or mild intermittent asthma/

7 1 or 2 or 3 or 4

8 5 and 6 and 7

For peer review only - http://bmjopen.bmj.com/site/about/guidelines.xhtml

BMJ Open

Validation of asthma recording in electronic health records: protocol for a systematic review

Journal:	*BMJ Open*
Manuscript ID	bmjopen-2016-014694.R1
Article Type:	Protocol
Date Submitted by the Author:	25-Jan-2017

Complete List of Authors:	Nissen, Francis; London School of Hygiene and Tropical Medicine, EPH - ENCD<br>Quint, Jennifer; Imperial College London, Respiratory Epidemiology, Occupational Medicine and Public Health<br>Wilkinson, Samantha; London School of Hygiene and Tropical Medicine, Epidemiology<br>Müllerová, Hana; GlaxoSmithKline, Epidemiology Department<br>Smeeth, Liam; London School of Hygiene and Tropical Medicine, Epidemiology and Population Health<br>Douglas, Ian; London School of Hygiene and Tropical Medicine, Epidemiology and Population Health

**Primary Subject Heading**:	Epidemiology
Secondary Subject Heading:	Respiratory medicine, Health informatics, Health services research
Keywords:	Asthma < THORACIC MEDICINE, Validation, Electronic Health Records, Sensitivity, Positive Predictive Value, Negative Predictive Value

SCHOLARONE™
Manuscripts

Validation of asthma recording in electronic health records: protocol for a systematic review

Authors: Francis Nissen,[1] Jennifer Quint,[2] Samantha Wilkinson,[1] Hana Mullerova,[3] Liam Smeeth,[1] Ian Douglas[1]

[1]Department of Non-Communicable Disease Epidemiology, London School of Hygiene and Tropical Medicine, London, UK; [2]National Heart and Lung Institute, Imperial College, London, UK; RWD & Epidemiology, GSK R&D, Uxbridge , UK

Correspondence:

Francis Nissen, MD, MSc, Department of Non-Communicable Disease Epidemiology, London School of Hygiene and Tropical Medicine, London, UK.

Address: Keppel Street, London, WC1E 7HT, UK

E-mail: francis.nissen@lshtm.ac.uk

For peer review only - http://bmjopen.bmj.com/site/about/guidelines.xhtml

ABSTRACT

Background: Asthma is a common, heterogeneous disease with significant morbidity and mortality worldwide. It can be difficult to define in epidemiological studies using electronic health records, as the diagnosis is based on non-specific respiratory symptoms and spirometry, neither of which are routinely registered. Electronic health records can nonetheless be valuable to study the epidemiology, management, health-care utilization and control of asthma. For health databases to be useful sources of information, asthma diagnoses should ideally be validated. The primary objectives are to provide an overview of the methods used to validate asthma diagnoses in electronic health records and summarise the results of the validation studies.

Methods: EMBASE and MEDLINE will be systematically searched for appropriate search terms. The searches will cover all studies in these databases up to October 2016 with no start date and will yield studies that have validated algorithms or codes for the diagnosis of asthma in electronic health records. At least one test validation measure (sensitivity, specificity, positive predictive value, negative predictive value or other) is necessary for inclusion. In addition, we require the validated algorithms to be compared to an external golden standard, such as a manual review, a questionnaire or an independent second database. We will summarise key data including author, year of publication, country, time period, date, data source, population, case characteristics, clinical events, algorithms, gold standard and

For peer review only - http://bmjopen.bmj.com/site/about/guidelines.xhtml

For peer review only

validation statistics in a uniform table.

Ethics and dissemination: This study is a synthesis of previously published studies and, therefore, no ethical approval is required. The results will be submitted to a peer-reviewed journal for publication. Results from this systematic review can be used to study outcome research on asthma and can be used to identify case definitions for asthma.

Trial registration number

The protocol has been registered in the PROSPERO database with registration number CRD42016041798.

Keywords

Asthma, Validation, Electronic Health Records, Sensitivity, Specificity, Positive Predictive Value, Negative Predictive Value

For peer review only

For peer review only - http://bmjopen.bmj.com/site/about/guidelines.xhtml

Strengths and limitations of this study

- To our knowledge, this is the first systematic review to identify and evaluate methods used to validate a recording of asthma diagnosis in electronic health records.
- The review of validation of asthma diagnosis in electronic health records could inform selection of asthma identification algorithms used by future health outcome studies and identify any gaps in quality and scope of validation studies. It will also provide an overview of the algorithms with their PPV, NPV, sensitivity or specificity.
- Different databases may validate different algorithms to identify asthma, which might limit the generalisability of these algorithms as they are context-specific.
- This review is focused on the methodology of asthma recording validation, and not on all outcome results of studies (except the validation results). Because of this, publication bias might be an issue (methods that do not find positive results may be less likely to have been published).

For peer review only

BACKGROUND

Asthma is a common chronic inflammatory disease of the airways. This condition is characterised by a variable expiratory airflow limitation which is generally reversible. The core symptoms are cough, wheeze, breathlessness and chest tightness [1]. Asthma episodes can range from mild attacks, which interrupt daily life and work productivity, to severe and life-threatening attacks [2]. Asthma is inherently variable and individuals will experience

For peer review only - http://bmjopen.bmj.com/site/about/guidelines.xhtml

fluctuating symptoms. Most commonly, asthma emerges in childhood, but it can also arise in adulthood. Therefore, adult asthma consists of both persistent or relapsed childhood disease and true incident adult disease. There is no cure, but with the right treatment, symptoms can usually be managed and asthma patients can lead their lives without disruption [1].

The widespread adoption of electronic health records (EHR) means that large population-based primary and secondary care databases are available, proving a great opportunity for research on asthma and other diseases. The availability of routinely generated longitudinal records for research has dramatically increased over the last decades [3]. However, the primary function of EHR is to support healthcare clinical decision making, not research purposes. The integrity of the research generated from EHR may be questionable, unless data are thoroughly validated for this purpose [4-7].

EHR are a digital reflection of the paper medical chart, while the main purpose of administrative claims data is administration of reimbursements to healthcare providers for their services. This systematic review will only consider data from EHR, as the quality measures between the two types of data can be markedly different [8 9].

EHR store information about diagnoses as clinical codes. A single code, or an algorithm consisting of multiple codes, can be used to retrieve records from EHR, and

For peer review only

For peer review only - http://bmjopen.bmj.com/site/about/guidelines.xhtml

additional restrictions can be applied such as age or exclusion of other diseases [7 10]. Alternatively, several authors have recently used natural language processing and machine learning techniques to automate algorithm generation for the identification of asthma diagnoses from large databases [11-13]. The most common method to assess the validity of algorithms is to compare them to a gold standard such as another linkable dataset or request a verification from the treating physician or the patient via a questionnaire [10]. Another approach is active case detection where the databases are constantly screened to identify cases that emerge [14].

Several limitations apply to the validation of diagnosis recording in EHR. First, individual databases often only cover a single care setting (primary or secondary care), as such case ascertainment only relies on a partial description of the healthcare pathway [15]. Another issue is that the validity of different diseases will not necessarily be the same in a given dataset. For example, mental health disorders such as anxiety or depression might be coded using less specific symptoms, whereas the validity of diagnoses with a very high specificity such as breast cancer is likely to be superior. There have been multiple studies which have measured the validity of specific databases for asthma [16 17]. Sharifi et al. have conducted a systematic review on validated methods to capture acute bronchospasm using

For peer review only - http://bmjopen.bmj.com/site/about/guidelines.xhtml

administrative or claims data [18], which yielded two validation studies of bronchospasm codes [11 19].

This systematic literature review aims to provide an overview of methods used to validate asthma diagnoses, specifically in EHR. Such a study has not yet been published in the medical literature, to the best of our knowledge.

Research question

The primary objectives of this systematic review are to provide an overview of both the methods with which asthma diagnosis recording has been validated in EHR and the estimates of the validation test measures.

The questions of interest for this systematic review are:

A. Which EHR have been used to obtain information on the diagnosis of asthma?

B. Which algorithms have been used to define an asthma diagnosis (including diagnostic codes, possible spirometry tests and clinical descriptions)?

C. How were the diagnostic criteria applied to the data sources and which other approaches have been used to validate a case definition?

D. What are the estimates for the PPV, NPV, specificity and sensitivity for a diagnosis of asthma in EHR?

METHODS

MEDLINE and EMBASE will be searched for the terms “asthma”, “validation”, “electronic

For peer review only

databases" and synonyms for each of these terms. In addition, reference lists of review articles and retrieved articles will be reviewed. The PRISMA flow diagram of this protocol, from Moher et al. [20], can be found in figure 1 and the search strategy can be found in the supplementary file.

Inclusion criteria

Any type of observational study design that used EHR to validate the recording of an asthma diagnosis will be considered. Articles will only be considered if published in English and before October 2016 without any specific start date. Within the databases, we will consider asthma diagnoses based on both structured data (such as laboratory results and prescriptions) and unstructured data (such as spirometry results). We require the validated algorithms to be compared to an external gold standard, such as a manual review, questionnaires (completed by the patient or their physician) or an independent second database. We will include algorithms formed of single codes, those requiring multiple case characteristics and algorithms generated by natural language processing or machine-learning.

Exclusion criteria

Studies which involve pharmacovigilance databases (signal detection or spontaneous reporting), studies without validation process of asthma recording and conference abstracts will be excluded. Algorithms used in databases originating from only claims data will also be excluded, as a systematic review on the validated methods to capture acute bronchospasm

For peer review only

For peer review only - http://bmjopen.bmj.com/site/about/guidelines.xhtml

using claims data has been published recently [18].

Two independent authors will scan the abstracts and titles against the research questions and exclusion criteria and select articles for full-text review. After this full-text article review, eligibility for inclusion in the report will be decided by consensus or arbitration by a third reviewer. A uniform table with information of each included study will be populated after data extraction, which will include information on the author, date of publication, journal, database, algorithms, population, gold standard and test measure(s).

Data synthesis

Studies and study data will be managed using EndNote and Microsoft Excel, respectively. The methods for asthma recording validation will be summarised in a narrative synthesis and tables describing all identified verification processes, and their results. These results will consist of the recorded PPV, NPV, sensitivity and specificity of the included studies. Where possible, these tests will be calculated if they are not reported within the study.

Dissemination and ethics

This study is a synthesis of previously published studies, so no ethical approval is required. The protocol has been registered in the PROSPERO database with registration number CRD42016041798. The results will be submitted for publication and will be disseminated through research conferences and peer reviewed journals.

For peer review only - http://bmjopen.bmj.com/site/about/guidelines.xhtml

Competing interests

FN and SW are funded by a GSK scholarship during their PhD programs.

Contributors

JQ, ID, LS and HM were responsible for developing the research question and have advised on the data collection and search strategies. FN drafted the manuscript, FN and SW will review the literature and summarise the found papers. ID is responsible for study management and coordination. All authors have read, commented on and approved the final manuscript.

Data sharing statement

Study data will be available on request to FN once the research team has completed pre-planned analyses.

For peer review only

References

1. WHO. Asthma Fact sheet No307. Secondary Asthma Fact sheet No307 2013.
2. GINA. Global Strategy for Asthma Management and Prevention 2016. Available from: www.ginasthma.org: Global Initiative for Asthma, 2016.
3. Langan SM, Benchimol EI, Guttmann A, et al. Setting the RECORD straight: developing a guideline for the REporting of studies Conducted using Observational Routinely collected Data. Clinical epidemiology 2013;**5**:29-31 doi: 10.2147/clep.s36885[published Online First: Epub Date]|.
4. Denney MJ, Long DM, Armistead MG, Anderson JL, Conway BN. Validating the extract, transform, load process used to populate a large clinical research database. International journal of medical informatics 2016;**94**:271-4 doi: 10.1016/j.ijmedinf.2016.07.009[published Online First: Epub Date]|.

For peer review only - http://bmjopen.bmj.com/site/about/guidelines.xhtml

5. Rimland JM, Abraha I, Luchetta ML, et al. Validation of chronic obstructive pulmonary disease (COPD) diagnoses in healthcare databases: a systematic review protocol. BMJ open 2016;**6**(6):e011777 doi: 10.1136/bmjopen-2016-011777[published Online First: Epub Date]|.

6. Abraha I, Giovannini G, Serraino D, Fusco M, Montedori A. Validity of breast, lung and colorectal cancer diagnoses in administrative databases: a systematic review protocol. BMJ open 2016;**6**(3):e010409 doi: 10.1136/bmjopen-2015-010409[published Online First: Epub Date]|.

7. Lo Re V, 3rd, Haynes K, Forde KA, Localio AR, Schinnar R, Lewis JD. Validity of The Health Improvement Network (THIN) for epidemiologic studies of hepatitis C virus infection. Pharmacoepidemiology and drug safety 2009;**18**(9):807-14 doi: 10.1002/pds.1784[published Online First: Epub Date]|.

8. Tang PC, Ralston M, Arrigotti MF, Qureshi L, Graham J. Comparison of Methodologies for Calculating Quality Measures Based on Administrative Data versus Clinical Data from an Electronic Health Record System: Implications for Performance Measures. Journal of the American Medical Informatics Association : JAMIA 2007;**14**(1):10-5 doi: 10.1197/jamia.M2198[published Online First: Epub Date]|.

9. Angier H, Gold R, Gallia C, et al. Variation in Outcomes of Quality Measurement by Data Source. Pediatrics 2014;**133**(6):e1676-82 doi: 10.1542/peds.2013-4277[published Online First: Epub Date]|.

10. Weiskopf NG, Weng C. Methods and dimensions of electronic health record data quality assessment: enabling reuse for clinical research. Journal of the American Medical Informatics Association : JAMIA 2013;**20**(1):144-51 doi: 10.1136/amiajnl-2011-000681[published Online First: Epub Date]|.

11. Sanders DL, Gregg W, Aronsky D. Identifying asthma exacerbations in a pediatric emergency department: a feasibility study. Int J Med Inform 2007;**76**(7):557-64 doi: 10.1016/j.ijmedinf.2006.03.003[published Online First: Epub Date]|.

12. Afzal Z, Engelkes M, Verhamme KMC, et al. Automatic generation of case-detection algorithms to identify children with asthma from large electronic health record databases. Pharmacoepidemiology and Drug Safety 2013;**22**(8):826-33 doi: http://dx.doi.org/10.1002/pds.3438[published Online First: Epub Date]|.

13. Wu ST, Juhn YJ, Sohn S, Liu H. Patient-level temporal aggregation for text-based asthma status ascertainment. J Am Med Inform Assoc 2014;**21**(5):876-84 doi: http://dx.doi.org/10.1136/amiajnl-2013-002463[published Online First: Epub Date]|.

14. Wing K, Douglas I, Bhaskaran K, et al. Development of predictive genetic tests for improving the safety of new medicines: the utilization of routinely collected electronic health records. Drug discovery today 2014;**19**(4):361-6 doi: 10.1016/j.drudis.2013.11.003[published Online First: Epub Date]|.

For peer review only - http://bmjopen.bmj.com/site/about/guidelines.xhtml

15. Wing K, Bhaskaran K, Smeeth L, et al. Optimising case detection within UK electronic health records: use of multiple linked databases for detecting liver injury. BMJ open 2016;**6**(9):e012102 doi: 10.1136/bmjopen-2016-012102[published Online First: Epub Date]|.

16. Xi N, Wallace R, Agarwal G, Chan D, Gershon A, Gupta S. Identifying patients with asthma in primary care electronic medical record systems: Chart analysis–based electronic algorithm validation study. Canadian Family Physician 2015;**61**(10):e474-e83

17. Cornish RP, Henderson J, Boyd AW, Granell R, Van Staa T, Macleod J. Validating childhood asthma in an epidemiological study using linked electronic patient records. BMJ Open 2014;**4**(4):e005345 doi: http://dx.doi.org/10.1136/bmjopen-2014-005345[published Online First: Epub Date]|.

18. Sharifi M, Krishanswami S, McPheeters ML. A systematic review of validated methods to capture acute bronchospasm using administrative or claims data. Vaccine 2013;**31 Suppl 10**:K12-20 doi: http://dx.doi.org/10.1016/j.vaccine.2013.06.091[published Online First: Epub Date]|.

19. Mullooly JP, Schuler R, Mesa J, Drew L, DeStefano F. Wheezing lower respiratory disease and vaccination of premature infants. Vaccine 2011;**29**(44):7611-7 doi: 10.1016/j.vaccine.2011.08.022[published Online First: Epub Date]|.

20. Moher D, Liberati A, Tetzlaff J, Altman DG. Preferred reporting items for systematic reviews and meta-analyses: the PRISMA Statement. Open medicine : a peer-reviewed, independent, open-access journal 2009;**3**(3):e123-30

For peer review only

For peer review only - http://bmjopen.bmj.com/site/about/guidelines.xhtml

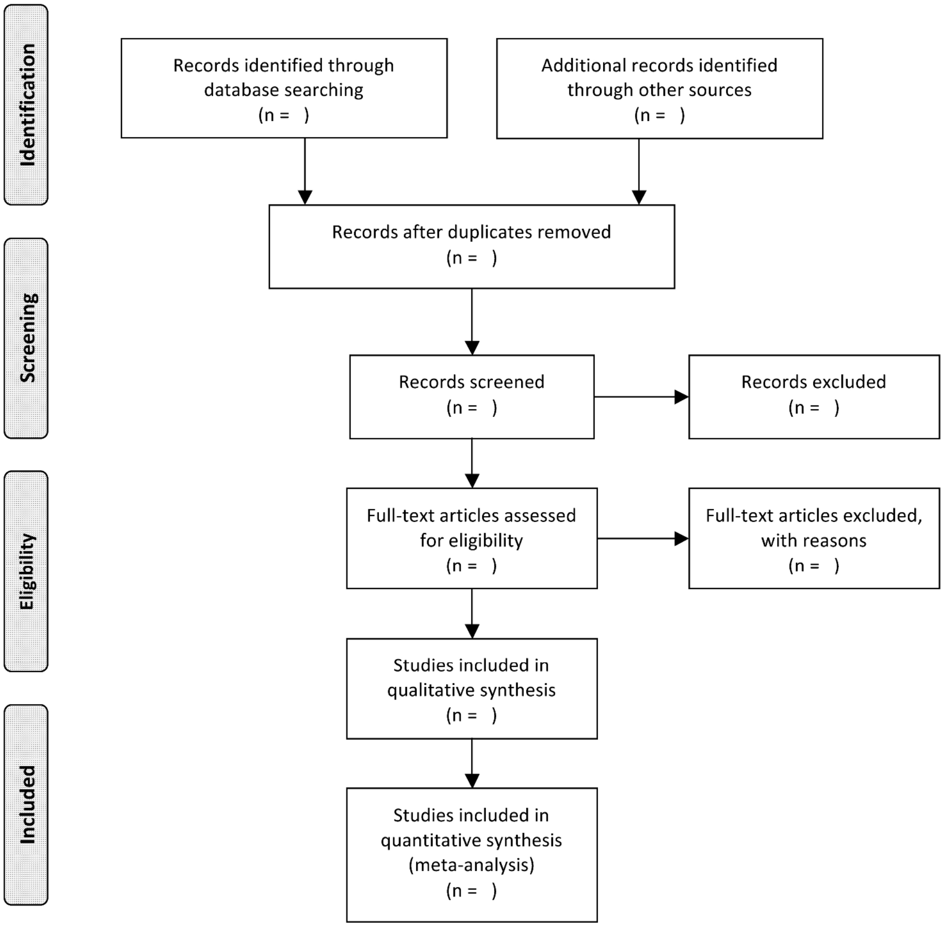

PRISMA flow diagram from Moher et al.

184x178mm (600 x 600 DPI)

Appendix 1: Algorithm used for literature review

Asthma validation in electronic health records: a systematic review

MEDLINE

1 (validat* or verif*).mp. [mp=title, abstract, original title, name of substance word, subject heading word, keyword heading word, protocol supplementary concept word, rare disease supplementary concept word, unique identifier]

2 (PPV or PNV or NPV or "positive predictive value*" or "negative predictive value*" or "predictive positive value*" or "predictive negative value*" or "likelihood ratio" or precision or accuracy or "receiver operating characteristic*" or ROC or kappa).mp. [mp=title, abstract, original title, name of substance word, subject heading word, keyword heading word, protocol supplementary concept word, rare disease supplementary concept word, unique identifier]

3 Validation Studies/ or validation.mp. or Validation Studies as Topic/

4 (electronic* or digital* or computeri?ed or programmed or automated or database or data base).mp. [mp=title, abstract, original title, name of substance word, subject heading word, keyword heading word, protocol supplementary concept word, rare disease supplementary concept word, unique identifier]

5 asthma.mp. or Asthma/ or Asthma, Occupational/ or Asthma, Exercise-Induced/

6 Database Management Systems/

7 1 or 2 or 3

8 4 or 6

9 5 and 7 and 8

For peer review only

For peer review only - http://bmjopen.bmj.com/site/about/guidelines.xhtml

EMBASE

1 (validat* or verif*).mp. [mp=title, abstract, heading word, drug trade name, original title, device manufacturer, drug manufacturer, device trade name, keyword]

2 validation.mp. or validation study/ or validation process/

3 (sensitivity or specificity or "Sensitivity and Specificity").mp. [mp=title, abstract, heading word, drug trade name, original title, device manufacturer, drug manufacturer, device trade name, keyword]

4 (PPV or PNV or NPV or "positive predictive value" or "predictive negative value" or "negative predictive value" or "likelihood ratio" or precision or accuracy or "receiver operating characteristic" or ROC or kappa).mp. [mp=title, abstract, heading word, drug trade name, original title, device manufacturer, drug manufacturer, device trade name, keyword, floating subheading]

5 (electronic* or digital* or computeri?ed or programmed or automated or database or data base).mp. [mp=title, abstract, heading word, drug trade name, original title, device manufacturer, drug manufacturer, device trade name, keyword]

6 mild persistent asthma/ or nocturnal asthma/ or experimental asthma/ or moderate persistent asthma/ or severe persistent asthma/ or Asthma.mp. or exercise induced asthma/ or occupational asthma/ or intrinsic asthma/ or asthma/ or allergic asthma/ or extrinsic asthma/ or mild intermittent asthma/

7 1 or 2 or 3 or 4

8 5 and 6 and 7

For peer review only

For peer review only - http://bmjopen.bmj.com/site/about/guidelines.xhtml

PRISMA-P (Preferred Reporting Items for Systematic review and Meta-Analysis Protocols) 2015 checklist: recommended items to address in a systematic review protocol*

Section and topic	Item No	Checklist item	Reported on Page #
**ADMINISTRATIVE INFORMATION**			
Title:			
Identification	1a	Identify the report as a protocol of a systematic review	1
Update	1b	If the protocol is for an update of a previous systematic review, identify as such	N/A
Registration	2	If registered, provide the name of the registry (such as PROSPERO) and registration number	3
Authors:			
Contact	3a	Provide name, institutional affiliation, e-mail address of all protocol authors; provide physical mailing address of corresponding author	1
Contributions	3b	Describe contributions of protocol authors and identify the guarantor of the review	10
Amendments	4	If the protocol represents an amendment of a previously completed or published protocol, identify as such and list changes; otherwise, state plan for documenting important protocol amendments	N/A
Support:			
Sources	5a	Indicate sources of financial or other support for the review	9
Sponsor	5b	Provide name for the review funder and/or sponsor	9
Role of sponsor or funder	5c	Describe roles of funder(s), sponsor(s), and/or institution(s), if any, in developing the protocol	9
**INTRODUCTION**			
Rationale	6	Describe the rationale for the review in the context of what is already known	6-7
Objectives	7	Provide an explicit statement of the question(s) the review will address with reference to participants, interventions, comparators, and outcomes (PICO)	N/A
**METHODS**			
Eligibility criteria	8	Specify the study characteristics (such as PICO, study design, setting, time frame) and report characteristics (such as years considered, language, publication status) to be used as criteria for eligibility for the review	8-9
Information sources	9	Describe all intended information sources (such as electronic databases, contact with study authors, trial registers or other grey literature sources) with planned dates of coverage	7
Search strategy	10	Present draft of search strategy to be used for at least one electronic database, including planned limits, such that it could be repeated	Uploaded separately

For peer review only

Study records:			
Data management	11a	Describe the mechanism(s) that will be used to manage records and data throughout the review	9
Selection process	11b	State the process that will be used for selecting studies (such as two independent reviewers) through each phase of the review (that is, screening, eligibility and inclusion in meta-analysis)	8-9
Data collection process	11c	Describe planned method of extracting data from reports (such as piloting forms, done independently, in duplicate), any processes for obtaining and confirming data from investigators	8-9
Data items	12	List and define all variables for which data will be sought (such as PICO items, funding sources), any pre-planned data assumptions and simplifications	9
Outcomes and prioritization	13	List and define all outcomes for which data will be sought, including prioritization of main and additional outcomes, with rationale	9
Risk of bias in individual studies	14	Describe anticipated methods for assessing risk of bias of individual studies, including whether this will be done at the outcome or study level, or both; state how this information will be used in data synthesis	4
Data synthesis	15a	Describe criteria under which study data will be quantitatively synthesised	8-9
	15b	If data are appropriate for quantitative synthesis, describe planned summary measures, methods of handling data and methods of combining data from studies, including any planned exploration of consistency (such as I^2, Kendall's τ)	8-9
	15c	Describe any proposed additional analyses (such as sensitivity or subgroup analyses, meta-regression)	N/A
	15d	If quantitative synthesis is not appropriate, describe the type of summary planned	9
Meta-bias(es)	16	Specify any planned assessment of meta-bias(es) (such as publication bias across studies, selective reporting within studies)	
Confidence in cumulative evidence	17	Describe how the strength of the body of evidence will be assessed (such as GRADE)	N/A

*** It is strongly recommended that this checklist be read in conjunction with the PRISMA-P Explanation and Elaboration (cite when available) for important clarification on the items. Amendments to a review protocol should be tracked and dated. The copyright for PRISMA-P (including checklist) is held by the PRISMA-P Group and is distributed under a Creative Commons Attribution Licence 4.0.**

From: Shamseer L, Moher D, Clarke M, Ghersi D, Liberati A, Petticrew M, Shekelle P, Stewart L, PRISMA-P Group. Preferred reporting items for systematic review and meta-analysis protocols (PRISMA-P) 2015: elaboration and explanation. BMJ. 2015 Jan 2;349(jan02 1):g7647.

For peer review only

BMJ Open

Validation of asthma recording in electronic health records: protocol for a systematic review

Journal:	*BMJ Open*
Manuscript ID	bmjopen-2016-014694.R2
Article Type:	Protocol
Date Submitted by the Author:	03-Apr-2017
Complete List of Authors:	Nissen, Francis; London School of Hygiene and Tropical Medicine, EPH - ENCD<br>Quint, Jennifer; Imperial College London, Respiratory Epidemiology, Occupational Medicine and Public Health<br>Wilkinson, Samantha; London School of Hygiene and Tropical Medicine, Epidemiology<br>Müllerová, Hana; GlaxoSmithKline, Epidemiology Department<br>Smeeth, Liam; London School of Hygiene and Tropical Medicine, Epidemiology and Population Health<br>Douglas, Ian; London School of Hygiene and Tropical Medicine, Epidemiology and Population Health
**Primary Subject Heading**:	Epidemiology
Secondary Subject Heading:	Respiratory medicine, Health informatics, Health services research
Keywords:	Asthma < THORACIC MEDICINE, Validation, Electronic Health Records, Sensitivity, Positive Predictive Value, Negative Predictive Value

SCHOLARONE™
Manuscripts

Validation of asthma recording in electronic health records: protocol for a systematic review

Authors: Francis Nissen,[1] Jennifer K Quint,[2] Samantha Wilkinson,[1] Hana Mullerova,[3] Liam Smeeth,[1] Ian J Douglas[1]

[1]Department of Non-Communicable Disease Epidemiology, London School of Hygiene and Tropical Medicine, London, UK; [2]National Heart and Lung Institute, Imperial College, London, UK; RWD & Epidemiology, GSK R&D, Uxbridge , UK

Correspondence:

Francis Nissen, MD, MSc, Department of Non-Communicable Disease Epidemiology, London School of Hygiene and Tropical Medicine, London, UK.

Address: Keppel Street, London, WC1E 7HT, UK

E-mail: francis.nissen@lshtm.ac.uk

For peer review only - http://bmjopen.bmj.com/site/about/guidelines.xhtml

ABSTRACT

Background: Asthma is a common, heterogeneous disease with significant morbidity and mortality worldwide. It can be difficult to define in epidemiological studies using electronic health records, as the diagnosis is based on non-specific respiratory symptoms and spirometry, neither of which are routinely registered. Electronic health records can nonetheless be valuable to study the epidemiology, management, health-care utilization and control of asthma. For health databases to be useful sources of information, asthma diagnoses should ideally be validated. The primary objectives are to provide an overview of the methods used to validate asthma diagnoses in electronic health records and summarise the results of the validation studies.

Methods: EMBASE and MEDLINE will be systematically searched for appropriate search terms. The searches will cover all studies in these databases up to October 2016 with no start date and will yield studies that have validated algorithms or codes for the diagnosis of asthma in electronic health records. At least one test validation measure (sensitivity, specificity, positive predictive value, negative predictive value or other) is necessary for inclusion. In addition, we require the validated algorithms to be compared to an external golden standard, such as a manual review, a questionnaire or an independent second database. We will summarise key data including author, year of publication, country, time period, date, data source, population, case characteristics, clinical events, algorithms, gold standard and

For peer review only - http://bmjopen.bmj.com/site/about/guidelines.xhtml

validation statistics in a uniform table.

Ethics and dissemination: This study is a synthesis of previously published studies and, therefore, no ethical approval is required. The results will be submitted to a peer-reviewed journal for publication. Results from this systematic review can be used to study outcome research on asthma and can be used to identify case definitions for asthma.

Trial registration number

The protocol has been registered in the PROSPERO database with registration number CRD42016041798.

Keywords

Asthma, Validation, Electronic Health Records, Sensitivity, Specificity, Positive Predictive Value, Negative Predictive Value

For peer review only

For peer review only - http://bmjopen.bmj.com/site/about/guidelines.xhtml

Strengths and limitations of this study

- To our knowledge, this is the first systematic review to identify and evaluate methods used to validate a recording of asthma diagnosis in electronic health records.
- The review of validation of asthma diagnosis in electronic health records could inform selection of asthma identification algorithms used by future health outcome studies and identify any gaps in quality and scope of validation studies. It will also provide an overview of the algorithms with their PPV, NPV, sensitivity or specificity.
- Different databases may validate different algorithms to identify asthma, which might limit the generalisability of these algorithms as they are context-specific.
- This review is focused on the methodology of asthma recording validation, and not on all outcome results of studies (except the validation results). Because of this, publication bias might be an issue (methods that do not find positive results may be less likely to have been published).

BACKGROUND

Asthma is a common chronic inflammatory disease of the airways. This condition is characterised by a variable expiratory airflow limitation which is generally reversible. The core symptoms are cough, wheeze, breathlessness and chest tightness [1]. Asthma episodes can range from mild attacks, which interrupt daily life and work productivity, to severe and life-threatening attacks [2]. Asthma is inherently variable and individuals will experience

For peer review only

For peer review only - http://bmjopen.bmj.com/site/about/guidelines.xhtml

fluctuating symptoms. Most commonly, asthma emerges in childhood, but it can also arise in adulthood. Therefore, adult asthma consists of both persistent or relapsed childhood disease and true incident adult disease. There is no cure, but with the right treatment, symptoms can usually be managed and asthma patients can lead their lives without disruption [1].

The widespread adoption of electronic health records (EHR) means that large population-based primary and secondary care databases are available, proving a great opportunity for research on asthma and other diseases. The availability of routinely generated longitudinal records for research has dramatically increased over the last decades [3]. However, the primary function of EHR is to support healthcare clinical decision making, not research purposes. The integrity of the research generated from EHR may be questionable, unless data are thoroughly validated for this purpose [4-7].

EHR are a digital reflection of the paper medical chart, while the main purpose of administrative claims data is administration of reimbursements to healthcare providers for their services. This systematic review will only consider data from EHR, as the quality measures between the two types of data can be markedly different [8 9].

EHR store information about diagnoses as clinical codes. A single code, or an algorithm consisting of multiple codes, can be used to retrieve records from EHR, and

For peer review only

For peer review only - http://bmjopen.bmj.com/site/about/guidelines.xhtml

additional restrictions can be applied such as age or exclusion of other diseases [7 10]. Alternatively, several authors have recently used natural language processing and machine learning techniques to automate algorithm generation for the identification of asthma diagnoses from large databases [11-13]. The most common method to assess the validity of algorithms is to compare them to a gold standard such as another linkable dataset or request a verification from the treating physician or the patient via a questionnaire [10]. Another approach is active case detection where the databases are constantly screened to identify cases that emerge [14].

Several limitations apply to the validation of diagnosis recording in EHR. First, individual databases often only cover a single care setting (primary or secondary care), as such case ascertainment only relies on a partial description of the healthcare pathway [15]. Another issue is that the validity of different diseases will not necessarily be the same in a given dataset. For example, mental health disorders such as anxiety or depression might be coded using less specific symptoms, whereas the validity of diagnoses with a very high specificity such as breast cancer is likely to be superior. There have been multiple studies which have measured the validity of specific databases for asthma [16 17]. Sharifi et al. have conducted a systematic review on validated methods to capture acute bronchospasm using

For peer review only - http://bmjopen.bmj.com/site/about/guidelines.xhtml

administrative or claims data [18], which yielded two validation studies of bronchospasm codes [11 19].

This systematic literature review aims to provide an overview of methods used to validate asthma diagnoses, specifically in EHR. Such a study has not yet been published in the medical literature, to the best of our knowledge.

Research question

The primary objectives of this systematic review are to provide an overview of both the methods with which asthma diagnosis recording has been validated in EHR and the estimates of the validation test measures.

The questions of interest for this systematic review are:

A. Which EHR that are not only based on claims data have been used to obtain information on the diagnosis of asthma?

B. Which algorithms have been used to define an asthma diagnosis (including diagnostic codes, possible spirometry tests and clinical descriptions)?

C. How were the diagnostic criteria applied to the data sources and which other approaches have been used to validate a case definition?

D. What are the estimates for the PPV, NPV, specificity and sensitivity for a diagnosis of asthma in EHR that are not solely claims-based?

METHODS

For peer review only - http://bmjopen.bmj.com/site/about/guidelines.xhtml

MEDLINE and EMBASE will be searched for the terms “asthma”, “validation”, “electronic databases” and synonyms for each of these terms. In addition, reference lists of review articles and retrieved articles will be reviewed. The PRISMA flow diagram of this protocol, from Moher et al. [20], can be found in figure 1 and the search strategy can be found in the supplementary file.

Inclusion criteria

Any type of observational study design that used EHR to validate the recording of an asthma diagnosis will be considered. Articles will only be considered if published in English and before October 2016 without any specific start date. Within the databases, we will consider asthma diagnoses based on both structured data (such as laboratory results and prescriptions) and free text data (such as spirometry results). We require the validated algorithms to be compared to an external gold standard, such as a manual review, questionnaires (completed by the patient or their physician) or an independent second database. We will include algorithms formed of single codes, those requiring multiple case characteristics and algorithms generated by natural language processing or machine-learning.

Exclusion criteria

Studies which involve pharmacovigilance databases (signal detection or spontaneous reporting), studies without validation process of asthma recording and conference abstracts will be excluded. Algorithms used in databases originating from only claims data will also be

For peer review only - http://bmjopen.bmj.com/site/about/guidelines.xhtml

excluded, as a systematic review on the validated methods to capture acute bronchospasm using claims data has been published recently [18].

Two independent authors will scan the abstracts and titles against the research questions and exclusion criteria and select articles for full-text review. After this full-text article review, eligibility for inclusion in the report will be decided by consensus or arbitration by a third reviewer. A uniform table with information of each included study will be populated after data extraction, which will include information on the author, date of publication, journal, database, algorithms, population, gold standard and test measure(s).

Data synthesis

Studies and study data will be managed using EndNote and Microsoft Excel, respectively. The methods for asthma recording validation will be summarised in a narrative synthesis and tables describing all identified verification processes, and their results. These results will consist of the recorded PPV, NPV, sensitivity and specificity of the included studies. Where possible, these tests will be calculated if they are not reported within the study.

Dissemination and ethics

This study is a synthesis of previously published studies, so no ethical approval is required. The protocol has been registered in the PROSPERO database with registration number CRD42016041798. The results will be submitted for publication and will be disseminated

For peer review only - http://bmjopen.bmj.com/site/about/guidelines.xhtml

For peer review only

through research conferences and peer reviewed journals.

Funding statement

This work was supported by GlaxoSmithKline (GSK), through a PhD scholarship for FN with grant number EPNCZF5310. The publishing of this study was supported by the Wellcome Trust: grant number 098504/Z/12/Z.

Competing interests

FN and SW are funded by a GSK scholarship during their PhD programs. JQ reports Grants from MRC, BLF, Wellcome Trust, and has received research funds from GSK, AZ, Quintiles IMS and had personal fees from AZ, Chiesi, BI. HM is an employee of GSK R&D and owns shares of GSK Plc. ID is funded by, holds stock in and has consulted for GSK.

Contributors

JQ, ID, LS and HM were responsible for developing the research question and have advised on the data collection and search strategies. FN drafted the manuscript, FN and SW will review the literature and summarise the found papers. ID is responsible for study management and coordination. All authors have read, commented on and approved the final manuscript.

For peer review only - http://bmjopen.bmj.com/site/about/guidelines.xhtml

Data sharing statement

Study data will be available on request to FN once the research team has completed pre-planned analyses.

Figure legend

1. Study screening process: PRISMA flow diagram from Moher et al.

Appendices

1. Search algorithms in the Embase and Medline databases
2. PRISMA P checklist 2015

References

1. WHO. Asthma Fact sheet No307. Secondary Asthma Fact sheet No307 2013.
2. GINA. Global Strategy for Asthma Management and Prevention 2016. Available from: www.ginasthma.org: Global Initiative for Asthma, 2016.
3. Langan SM, Benchimol EI, Guttmann A, et al. Setting the RECORD straight: developing a guideline for the REporting of studies Conducted using Observational Routinely collected Data. Clinical epidemiology 2013;**5**:29-31 doi: 10.2147/clep.s36885[published Online First: Epub Date]|.
4. Denney MJ, Long DM, Armistead MG, Anderson JL, Conway BN. Validating the extract, transform, load process used to populate a large clinical research database. International journal of medical informatics 2016;**94**:271-4 doi: 10.1016/j.ijmedinf.2016.07.009[published Online First: Epub Date]|.
5. Rimland JM, Abraha I, Luchetta ML, et al. Validation of chronic obstructive pulmonary disease (COPD) diagnoses in healthcare databases: a systematic review protocol. BMJ open 2016;**6**(6):e011777 doi: 10.1136/bmjopen-2016-011777[published Online First: Epub Date]|.
6. Abraha I, Giovannini G, Serraino D, Fusco M, Montedori A. Validity of breast, lung and colorectal cancer diagnoses in administrative databases: a systematic review protocol. BMJ open 2016;**6**(3):e010409 doi: 10.1136/bmjopen-2015-010409[published Online First: Epub Date]|.

For peer review only - http://bmjopen.bmj.com/site/about/guidelines.xhtml

7. Lo Re V, 3rd, Haynes K, Forde KA, Localio AR, Schinnar R, Lewis JD. Validity of The Health Improvement Network (THIN) for epidemiologic studies of hepatitis C virus infection. Pharmacoepidemiology and drug safety 2009;**18**(9):807-14 doi: 10.1002/pds.1784[published Online First: Epub Date]|.

8. Tang PC, Ralston M, Arrigotti MF, Qureshi L, Graham J. Comparison of Methodologies for Calculating Quality Measures Based on Administrative Data versus Clinical Data from an Electronic Health Record System: Implications for Performance Measures. Journal of the American Medical Informatics Association : JAMIA 2007;**14**(1):10-5 doi: 10.1197/jamia.M2198[published Online First: Epub Date]|.

9. Angier H, Gold R, Gallia C, et al. Variation in Outcomes of Quality Measurement by Data Source. Pediatrics 2014;**133**(6):e1676-82 doi: 10.1542/peds.2013-4277[published Online First: Epub Date]|.

10. Weiskopf NG, Weng C. Methods and dimensions of electronic health record data quality assessment: enabling reuse for clinical research. Journal of the American Medical Informatics Association : JAMIA 2013;**20**(1):144-51 doi: 10.1136/amiajnl-2011-000681[published Online First: Epub Date]|.

11. Sanders DL, Gregg W, Aronsky D. Identifying asthma exacerbations in a pediatric emergency department: a feasibility study. Int J Med Inform 2007;**76**(7):557-64 doi: 10.1016/j.ijmedinf.2006.03.003[published Online First: Epub Date]|.

12. Afzal Z, Engelkes M, Verhamme KMC, et al. Automatic generation of case-detection algorithms to identify children with asthma from large electronic health record databases. Pharmacoepidemiology and Drug Safety 2013;**22**(8):826-33 doi: http://dx.doi.org/10.1002/pds.3438[published Online First: Epub Date]|.

13. Wu ST, Juhn YJ, Sohn S, Liu H. Patient-level temporal aggregation for text-based asthma status ascertainment. J Am Med Inform Assoc 2014;**21**(5):876-84 doi: http://dx.doi.org/10.1136/amiajnl-2013-002463[published Online First: Epub Date]|.

14. Wing K, Douglas I, Bhaskaran K, et al. Development of predictive genetic tests for improving the safety of new medicines: the utilization of routinely collected electronic health records. Drug discovery today 2014;**19**(4):361-6 doi: 10.1016/j.drudis.2013.11.003[published Online First: Epub Date]|.

15. Wing K, Bhaskaran K, Smeeth L, et al. Optimising case detection within UK electronic health records: use of multiple linked databases for detecting liver injury. BMJ open 2016;**6**(9):e012102 doi: 10.1136/bmjopen-2016-012102[published Online First: Epub Date]|.

16. Xi N, Wallace R, Agarwal G, Chan D, Gershon A, Gupta S. Identifying patients with asthma in primary care electronic medical record systems: Chart analysis–based electronic algorithm validation study. Canadian Family Physician 2015;**61**(10):e474-e83

17. Cornish RP, Henderson J, Boyd AW, Granell R, Van Staa T, Macleod J. Validating childhood

For peer review only

For peer review only - http://bmjopen.bmj.com/site/about/guidelines.xhtml

asthma in an epidemiological study using linked electronic patient records. BMJ Open 2014;**4**(4):e005345 doi: http://dx.doi.org/10.1136/bmjopen-2014-005345[published Online First: Epub Date]|.

18. Sharifi M, Krishanswami S, McPheeters ML. A systematic review of validated methods to capture acute bronchospasm using administrative or claims data. Vaccine 2013;**31 Suppl 10**:K12-20 doi: http://dx.doi.org/10.1016/j.vaccine.2013.06.091[published Online First: Epub Date]|.
19. Mullooly JP, Schuler R, Mesa J, Drew L, DeStefano F. Wheezing lower respiratory disease and vaccination of premature infants. Vaccine 2011;**29**(44):7611-7 doi: 10.1016/j.vaccine.2011.08.022[published Online First: Epub Date]|.
20. Moher D, Liberati A, Tetzlaff J, Altman DG. Preferred reporting items for systematic reviews and meta-analyses: the PRISMA Statement. Open medicine : a peer-reviewed, independent, open-access journal 2009;**3**(3):e123-30

For peer review only

For peer review only - http://bmjopen.bmj.com/site/about/guidelines.xhtml

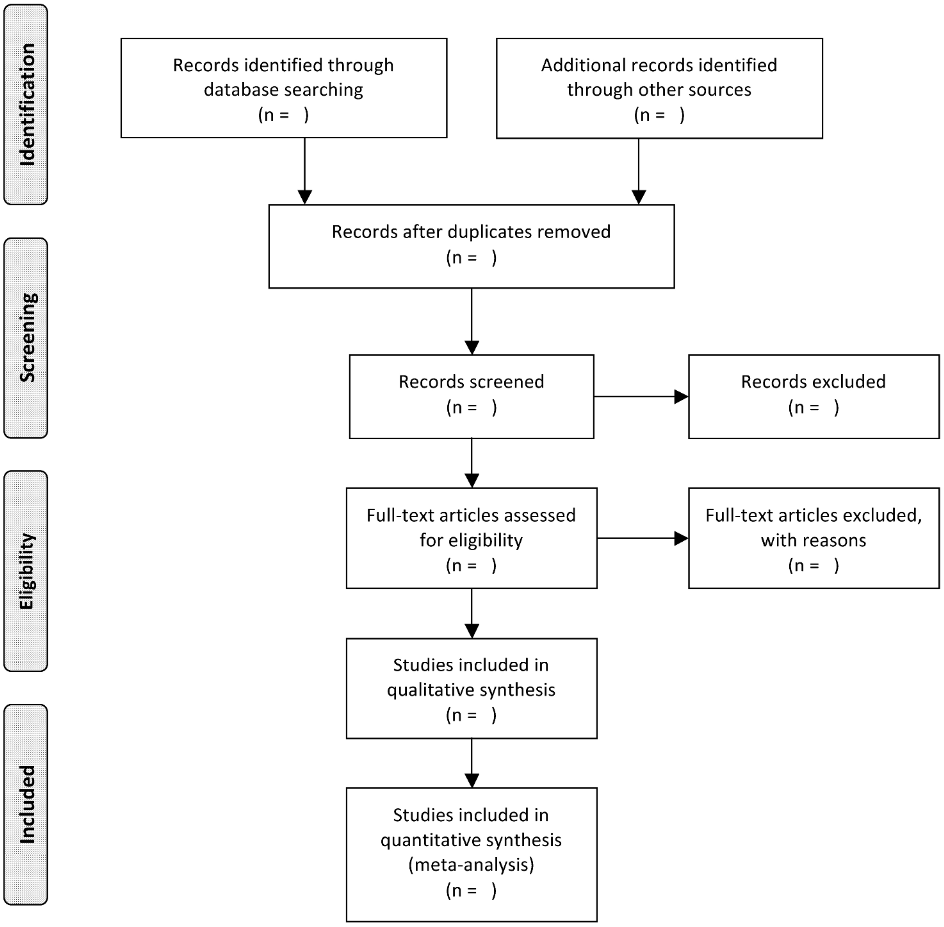

1.Study screening process: PRISMA flow diagram from Moher et al.

184x178mm (600 x 600 DPI)

Appendix 1: Algorithm used for literature review

Asthma validation in electronic health records: a systematic review

MEDLINE

1 (validat* or verif*).mp. [mp=title, abstract, original title, name of substance word, subject heading word, keyword heading word, protocol supplementary concept word, rare disease supplementary concept word, unique identifier]

2 (PPV or PNV or NPV or "positive predictive value*" or "negative predictive value*" or "predictive positive value*" or "predictive negative value*" or "likelihood ratio" or precision or accuracy or "receiver operating characteristic*" or ROC or kappa).mp. [mp=title, abstract, original title, name of substance word, subject heading word, keyword heading word, protocol supplementary concept word, rare disease supplementary concept word, unique identifier]

3 Validation Studies/ or validation.mp. or Validation Studies as Topic/

4 (electronic* or digital* or computeri?ed or programmed or automated or database or data base).mp. [mp=title, abstract, original title, name of substance word, subject heading word, keyword heading word, protocol supplementary concept word, rare disease supplementary concept word, unique identifier]

5 asthma.mp. or Asthma/ or Asthma, Occupational/ or Asthma, Exercise-Induced/

6 Database Management Systems/

7 1 or 2 or 3

8 4 or 6

9 5 and 7 and 8

For peer review only

For peer review only - http://bmjopen.bmj.com/site/about/guidelines.xhtml

EMBASE

1 (validat* or verif*).mp. [mp=title, abstract, heading word, drug trade name, original title, device manufacturer, drug manufacturer, device trade name, keyword]

2 validation.mp. or validation study/ or validation process/

3 (sensitivity or specificity or "Sensitivity and Specificity").mp. [mp=title, abstract, heading word, drug trade name, original title, device manufacturer, drug manufacturer, device trade name, keyword]

4 (PPV or PNV or NPV or "positive predictive value" or "predictive negative value" or "negative predictive value" or "likelihood ratio" or precision or accuracy or "receiver operating characteristic" or ROC or kappa).mp. [mp=title, abstract, heading word, drug trade name, original title, device manufacturer, drug manufacturer, device trade name, keyword, floating subheading]

5 (electronic* or digital* or computeri?ed or programmed or automated or database or data base).mp. [mp=title, abstract, heading word, drug trade name, original title, device manufacturer, drug manufacturer, device trade name, keyword]

6 mild persistent asthma/ or nocturnal asthma/ or experimental asthma/ or moderate persistent asthma/ or severe persistent asthma/ or Asthma.mp. or exercise induced asthma/ or occupational asthma/ or intrinsic asthma/ or asthma/ or allergic asthma/ or extrinsic asthma/ or mild intermittent asthma/

7 1 or 2 or 3 or 4

8 5 and 6 and 7

For peer review only

For peer review only - http://bmjopen.bmj.com/site/about/guidelines.xhtml

PRISMA-P (Preferred Reporting Items for Systematic review and Meta-Analysis Protocols) 2015 checklist: recommended items to address in a systematic review protocol*

Section and topic	Item No	Checklist item	Reported on Page #
**ADMINISTRATIVE INFORMATION**			
Title:			
Identification	1a	Identify the report as a protocol of a systematic review	1
Update	1b	If the protocol is for an update of a previous systematic review, identify as such	N/A
Registration	2	If registered, provide the name of the registry (such as PROSPERO) and registration number	3
Authors:			
Contact	3a	Provide name, institutional affiliation, e-mail address of all protocol authors; provide physical mailing address of corresponding author	1
Contributions	3b	Describe contributions of protocol authors and identify the guarantor of the review	10
Amendments	4	If the protocol represents an amendment of a previously completed or published protocol, identify as such and list changes; otherwise, state plan for documenting important protocol amendments	N/A
Support:			
Sources	5a	Indicate sources of financial or other support for the review	9
Sponsor	5b	Provide name for the review funder and/or sponsor	9
Role of sponsor or funder	5c	Describe roles of funder(s), sponsor(s), and/or institution(s), if any, in developing the protocol	9
**INTRODUCTION**			
Rationale	6	Describe the rationale for the review in the context of what is already known	6-7
Objectives	7	Provide an explicit statement of the question(s) the review will address with reference to participants, interventions, comparators, and outcomes (PICO)	N/A
**METHODS**			
Eligibility criteria	8	Specify the study characteristics (such as PICO, study design, setting, time frame) and report characteristics (such as years considered, language, publication status) to be used as criteria for eligibility for the review	8-9
Information sources	9	Describe all intended information sources (such as electronic databases, contact with study authors, trial registers or other grey literature sources) with planned dates of coverage	7
Search strategy	10	Present draft of search strategy to be used for at least one electronic database, including planned limits, such that it could be repeated	Uploaded separately

For peer review only

Study records:			
Data management	11a	Describe the mechanism(s) that will be used to manage records and data throughout the review	9
Selection process	11b	State the process that will be used for selecting studies (such as two independent reviewers) through each phase of the review (that is, screening, eligibility and inclusion in meta-analysis)	8-9
Data collection process	11c	Describe planned method of extracting data from reports (such as piloting forms, done independently, in duplicate), any processes for obtaining and confirming data from investigators	8-9
Data items	12	List and define all variables for which data will be sought (such as PICO items, funding sources), any pre-planned data assumptions and simplifications	9
Outcomes and prioritization	13	List and define all outcomes for which data will be sought, including prioritization of main and additional outcomes, with rationale	9
Risk of bias in individual studies	14	Describe anticipated methods for assessing risk of bias of individual studies, including whether this will be done at the outcome or study level, or both; state how this information will be used in data synthesis	4
Data synthesis	15a	Describe criteria under which study data will be quantitatively synthesised	8-9
	15b	If data are appropriate for quantitative synthesis, describe planned summary measures, methods of handling data and methods of combining data from studies, including any planned exploration of consistency (such as I^2, Kendall's τ)	8-9
	15c	Describe any proposed additional analyses (such as sensitivity or subgroup analyses, meta-regression)	N/A
	15d	If quantitative synthesis is not appropriate, describe the type of summary planned	9
Meta-bias(es)	16	Specify any planned assessment of meta-bias(es) (such as publication bias across studies, selective reporting within studies)	
Confidence in cumulative evidence	17	Describe how the strength of the body of evidence will be assessed (such as GRADE)	N/A

*** It is strongly recommended that this checklist be read in conjunction with the PRISMA-P Explanation and Elaboration (cite when available) for important clarification on the items. Amendments to a review protocol should be tracked and dated. The copyright for PRISMA-P (including checklist) is held by the PRISMA-P Group and is distributed under a Creative Commons Attribution Licence 4.0.**

From: Shamseer L, Moher D, Clarke M, Ghersi D, Liberati A, Petticrew M, Shekelle P, Stewart L, PRISMA-P Group. Preferred reporting items for systematic review and meta-analysis protocols (PRISMA-P) 2015: elaboration and explanation. BMJ. 2015 Jan 2;349(jan02 1):g7647.

For peer review only
